# Ligand-Baited Proteomics Reveals Vesicle Trafficking Proteins as Putative Site of Action of Indaziflam

**DOI:** 10.1021/acs.jafc.6c07779

**Published:** 2026-10-09

**Authors:** Mohit Mahey, Peter Knut Lundquist, Eric Lloyd Patterson

**Affiliations:** † Department of Plant, Soil, and Microbial Sciences, 3078 Michigan State University, East Lansing, Michigan 48824, United States; ‡ Department of Biochemistry & Molecular Biology, Michigan State University, East Lansing, Michigan 48824, United States; § Plant Resilience Institute, Michigan State University, East Lansing, Michigan 48824, United States

**Keywords:** Arabidopsis thaliana, Oryza sativa, indaziflam

## Abstract

Indaziflam is a potent herbicide that has become an important tool for pre-emergence weed control. Although it is classified as a cellulose biosynthesis inhibitor, its precise site of action remains unresolved. Here, we show that indaziflam preferentially affects the root elongation zone, where cells exhibit swelling and frequent rupture. We performed ligand-baited coprecipitation assays using root tissue lysates from *Arabidopsis thaliana* and *Oryza sativa*. Proteomic analysis revealed that the highly enriched proteins were associated with vesicular trafficking, including multiple coatomer subunits, while no cellulose synthase components were detected. Live-cell imaging using FM4-64 dye demonstrated that indaziflam does not cause a complete inhibition of clathrin-mediated endocytosis. AlphaFold3-based protein–ligand modeling further identified relatively stable predicted indaziflam poses with select trafficking-associated proteins, including coatomer subunit β. Collectively, our results support a model in which indaziflam indirectly disrupts cellulose biosynthesis by interfering with intracellular trafficking processes required for proper cellulose synthase complex homeostasis.

## Graphical abstract

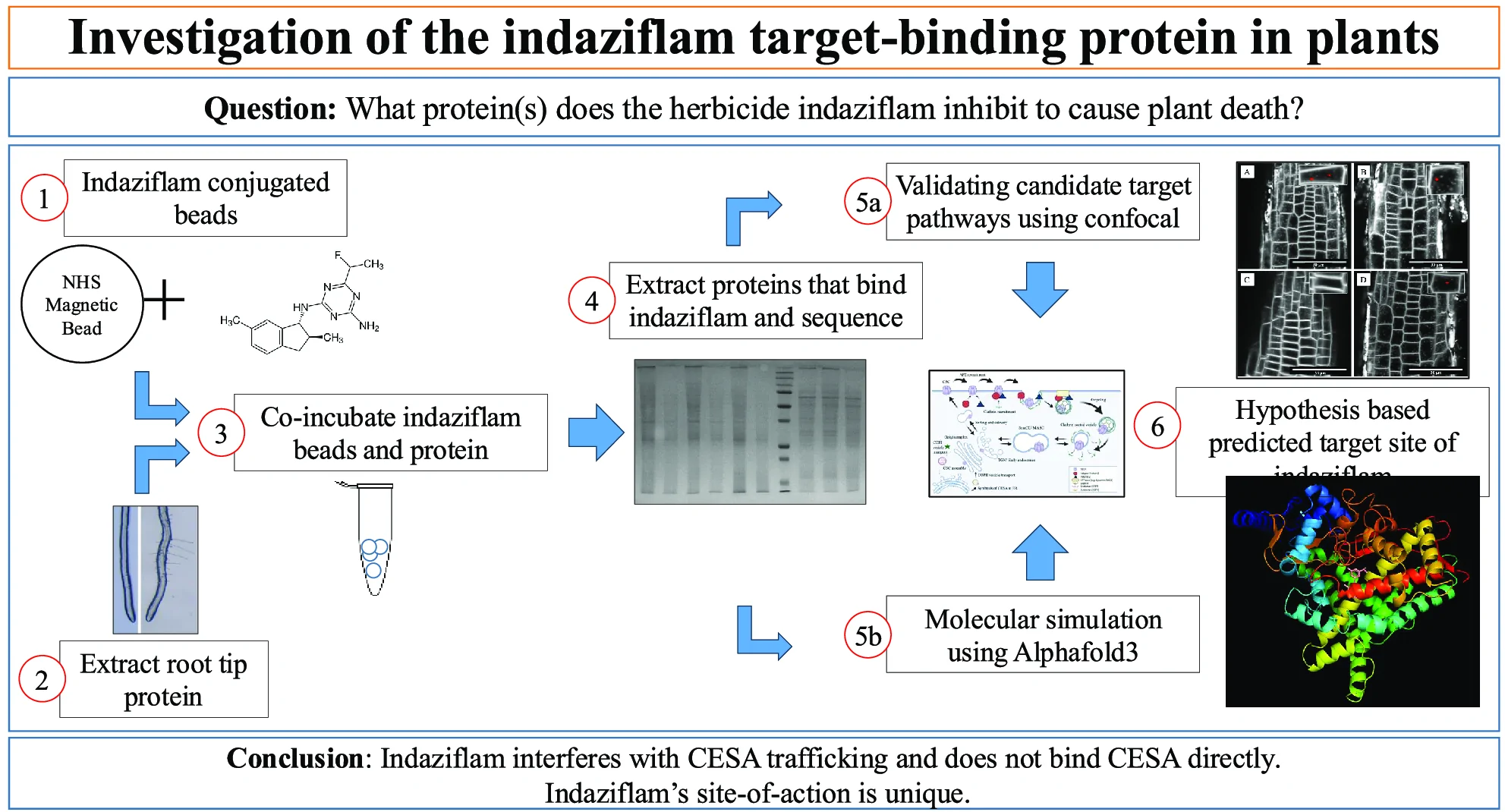

## Introduction

Herbicides are the primary tool used in modern conventional agriculture for the management of weeds. Since the discovery of the herbicide 2,4-D in the 1940s, hundreds of novel herbicidal compounds have been developed and commercialized. The vast majority of herbicides act by directly targeting specific proteins, acting as inhibitors and disrupting essential cellular processes or metabolic pathways.^1^ These protein targets are collectively referred to as the herbicide’s mechanism of action, with the specific binding pocket known as the site of action (SOA). While the SOA has been clearly identified for most herbicides, for some that elicit atypical physiological symptoms or that have been more recently introduced, they remain ambiguous.

′The herbicide indaziflam (N-[(1*R*,2*S*)-2,3-dihydro-2,6-dimethyl-1H-inden-1-yl]-6-[(1RS)-1-fluoroethyl]-1,3,5-triazine-2,4-diamine) (Figure 1) is applied before weed seed germination (a preapplied herbicide) and, in some cases, early postemergence. Indaziflam provides control of an extremely broad spectrum of annual monocotyledonous and dicotyledonous weed species. As it primarily affects seed germination, perennial crop systems with annual weed problems such as turfgrass, forestry systems, ornamental plantings, rangelands, and industrial vegetation are ideal systems. In 2010, indaziflam was registered for turf use by the U.S. Environmental Protection Agency. Indaziflam is also an exceptionally potent compound, exhibiting biological activity at picomolar to nanomolar concentrations and in some cases providing multiyear control of weed seed germination.^2,3^ To date, only limited field-evolved resistance to indaziflam has been reported, potentially reflecting its restricted use patterns in orchards, turfgrass, rangelands, and other perennial production systems.^4,5^

**Figure 1:**
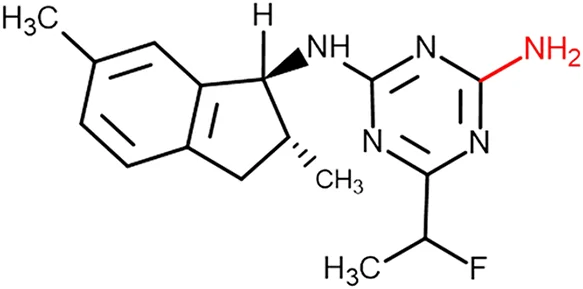
Molecular structure of indaziflam. The primary amine used for cross-linking to beads is highlighted in red.

Indaziflam has been provisionally classified as a cellulose biosynthesis inhibitor (CBI; HRAC Group 29) based on its pronounced effects on cell wall formation within root meristematic and elongation zones.^2^ The plant cell wall is a complex macromolecular structure composed of cellulose, hemicellulose, other polysaccharides, and structural proteins, implicating numerous coordinated proteins as potential SOAs. Cellulose, the primary load-bearing component of the cell wall, consists of β-1,4-linked glucan chains and is essential for cell division, elongation, and overall plant growth. In higher plants, cellulose is synthesized at the plasma membrane by cellulose synthase enzymes (CESAs), which assemble into cellulose synthase complexes (CSCs) composed of three distinct CESA isoforms. In addition to CESAs, multiple accessory proteins (e.g., KORRIGAN,^6^ COBRA,^7^ and CELLULOSE SYNTHASE INTERACTING1 (CSI1)^8^) are required for proper cellulose production and deposition. Other regulatory proteins further modulate CSC dynamics through controlled CESA trafficking, including endocytosis, exocytosis, and post-translational modification.^9,10^

Unlike other CBIs, such as isoxaben and flupoxam, which exhibit selective phytotoxicity, primarily toward dicotyledonous species, indaziflam displays activity across both monocotyledonous and dicotyledonous plants.^11^ This broader spectrum suggests that indaziflam inhibits cellulose biosynthesis at a slightly different SOA, distinct from previously characterized CBIs. Multiple other lines of evidence support this hypothesis. Isoxaben and related CBIs are known to directly target CESA proteins^12–14^ with several *Arabidopsis thaliana* mutants harboring specific target-site substitutions in CESA genes causing isoxaben resistance; however, these mutations do not confer cross-resistance to indaziflam.^2^ Genetic screening in *A. thaliana* has identified mutant lines exhibiting only mild resistance to indaziflam, including mutations in *CESA6* (E929 K, G935E, D605N, and S360N), which display modest but statistically significant differences in root growth at 250 pM indaziflam.^15^ Additionally, mutations in *CULLIN1 (CUL1)* have been reported to reduce sensitivity to indaziflam at 400 pM.^16^ Notably, no mutant has been identified that exhibits tolerance at concentrations lethal to wild-type plants, suggesting that indaziflam may act on multiple targets, or that mutations disrupting its primary binding site are lethal and therefore not recoverable.

Further evidence of a unique mode of inhibition arises from live-cell imaging studies. Fluorescently labeled *A. thaliana* lines demonstrate that indaziflam alters CSC behavior in a manner distinct from isoxaben.^17^ Whereas isoxaben rapidly depletes CSCs from the plasma membrane focal plane, indaziflam causes CSC accumulation at the membrane while significantly reducing their average velocity, indicating a fundamentally different mechanism of cellulose biosynthesis disruption.^2,18,19^

Despite these observations, the direct molecular target(s) of indaziflam remains(s) unidentified, and its precise cellular consequences are not fully understood. Elucidating the SOA of indaziflam is critical for optimizing its use in integrated weed management, mitigating the risk of resistance evolution, and informing the rational development of next-generation herbicidal chemistries. Moreover, identifying the molecular target(s) of indaziflam will aid in the discovery of resistance-conferring mutations and enable the potential engineering of indaziflam-resistant crop and turfgrass species. In this study, we employ untargeted native protein pull-down assays to identify candidate indaziflam-binding proteins and investigate their effects on clathrin-mediated endocytosis using confocal microscopy. Structural modeling of candidate targets using AlphaFold3 ^20^ further provides further insights into potential binding sites and mechanisms of action for indaziflam.

## Materials and Methods

### Plant Material and Growth Conditions

*A. thaliana* (Col-0) and *O. sativa* (cv. Nipponbare) were used as representative dicot and monocot species for pull-down assays. All chemicals were of analytical reagent grade and purchased from Sigma-Aldrich, St. Louis, MO, unless specified. Technical grade indaziflam (Bayer Crop Science, MO) (Figure 1) was kindly provided by Bayer, and dissolved in 100% dimethyl sulfoxide (DMSO, Mallinckrodt, Paris, Kentucky) at a final concentration of 20 mM as a stock solution. Another 0.4% (v/v) *N*,*N*-diethylethanamine (TEA) solution was made by adding into the stock indaziflam DMSO solution. Sodium dodecyl sulfate (SDS) was prepared as a 10% stock solution. MilliporePureProteome NHS FlexiBind Magnetic Bead System was used in this research. For confocal microscopy assay, *A. thaliana* (Col-0) seeds were surface-sterilized by immersion in 70% ethanol for 3 min, followed by treatment with 10% sodium hypochlorite or bleach (Clorox company, CA) containing 0.01% Triton X-100 for 20 min, and subsequently rinsed three times with sterile distilled water. Sterilized seeds were sown on vertical square (10 × 10 cm^2^) Petri plates containing 1% agar (Thermo Scientific Inc., MA) supplemented with half-strength Murashige & Skoog (M519) basal medium (Caisson lab science, UT). Plates were vernalized at 4 °C for 2 days to synchronize germination and then transferred to a growth chamber maintained at 25 °C during a 16-h photoperiod and 21 °C during an 8-h dark period for 5 days, with the photosynthetically active radiation at 150 μmol m^–2^ s^–1^.

### Cellular Morphology of *A. thaliana* Roots Treated with Indaziflam

Four-day-old *A. thaliana* seedlings were treated with 0.1% (v/v) DMSO as control and 8000 nM indaziflam for 2 h. After 2 h of treatment, the seedlings were incubated with 4 μM FM4-64 SynaptoRed C2 (Biotium Inc., CA) for 5 min at room temperature, followed by three washes in half-strength Murashige & Skoog (M519) basal medium and distilled water to remove excess dye. Data were collected with a confocal microscope using an upright Olympus Fluoview spectral FV1000 confocal microscope with a UPLAPO 40× oil-immersion objective (NA 0.85) and pictures were processed with FIJI (v2.16.0 ^21^).

To phenotype root morphology under indaziflam treatment, *A. thaliana* seeds and agar media were prepared as described above, except a different amount of indaziflam was provided in the media, with the final concentration of 0 pM, 200 pM, 400 pM, 500 pM, and 600 pM. Root hairs were photographed 12 days after indaziflam treatment, using the stereomicroscope Nikon SMZ 800N with C-W10×B/22 eyepieces. Pictures were processed with NIS-Elements Viewer (v5.21.00).

### Indaziflam Extinction Coefficient Measurement

The extinction coefficient of indaziflam in DMSO was measured to facilitate rapid and accurate quantification. A series of indaziflam-DMSO solutions were prepared, with concentrations 16.65 μM, 33.3 μM, 66.6 μM, 133.2 μM, 199.8 μM, and 266.4 μM by dilution from the 3.33 mM stock solution. For each concentration, the absorbance values were measured with an ultraviolet spectrophotometer (Thermo Scientific GENESYS) at 280 nm wavelength, using quartz cuvettes, at room temperature. A 100% DMSO solution was used as the blank control. Using 3 replicates per concentration, a standard curve was established (Figure S1), which was used to calculate indaziflam’s extinction coefficient.

The extinction coefficient per mole of indaziflam described below is the value at the first absorption peak position. It was calculated using Beer–Lambert’s law.

A=ϵCL

In the equation above, *A* is the absorbance at 280 nm for indaziflam solutions. *C* is the molar concentration (mol L^–1^) of each indaziflam sample. *L* is the path length (cm) used for recording the absorption spectrum. In our experiments, *L* was fixed at 1 cm. ε is the extinction coefficient per mole of indaziflam (L mol^–1^ cm^–1^) in the solvent (DMSO).

### Membrane Protein Solubilization Buffer and Quenched Bead Preparation

As the mature CESA is located primarily in the plasma membrane (PM), many proteins associated with CESA are lipophilic and/or membrane bound. To ensure efficient extraction of these proteins and to eliminate nonspecific aggregation associations, we used a protein extraction buffer developed previously.^22^ Membrane protein solubilization buffer was made containing 100 mM Tris-HCl at pH 7.3, 150 mM NaCl, 10% glycerol, 20 mM NaF, 1% Triton X-100, and complete protease inhibitor cocktail 1x (Roche, Basel, Switzerland).^22^ The buffer was aliquoted and stored at −20 °C. Quenched beads were prepared for preclearing protein extracts and negative controls (flow through). Following the manufacturer’s instructions, bead slurry was separated from storage buffer, washed with membrane protein solubilization buffer three times, quenched, resuspended in wash/coupling buffer (phosphate buffered saline, pH = 7.4), and stored at 4 °C until use.

### Indaziflam Conjugation to NHS Beads

Indaziflam was conjugated to NHS FlexiBind magnetic beads via its free primary amine group conjugated to the carboxy group on the beads according to the manufacturer’s instructions. In brief, bead slurry was removed from storage and washed with 1 mM cold HCl solution. Indaziflam dissolved in DMSO (20 mM) with 0.4% (v/v) TEA was added to the beads (functional group concentration >3.4 mM), and then the mixture was incubated with continuous mixing on a rotisserie at room temperature overnight (12–16 h).^23^ The next day, beads were quenched, resuspended in 100 μL wash/coupling buffer (PBS), and then stored at 4 °C until use. Coupling efficiency was determined by subtracting the amount of unbound indaziflam from the amount reacted with the beads, using absorbance at 280 nm and the extinction coefficient we previously determined. The confirmation was completed by analysis of a series of indaziflam solutions which were prepared by the dilution of a 3.33 mM indaziflam stock solution. Conjugation efficiency was calculated by dividing the molar amount of indaziflam retained by the beads by the minimal molar amount of NHS-ester groups on the beads (Table 1).

**Table 1:** Indaziflam Conjugation Rates with NHS Magnetic Beads

	indaziflam conc. (mM)				
root lysate source	before conjugation	after conjugation	estimated indaziflam bound to beads^a^ (mM)	minimum concentration of bead binding sites (mM)	indaziflam conjugation rate^b^ (%)
*A. thaliana*	21.03 ± 1.3	17.74 ± 0.3	3.29 ± 0.3	3.4	96.8 ± 8.5
*O. sativa* subsp. japonica	19.72 ± 0.5	14.07 ± 0.7	5.65 ± 0.7		166 ± 21

a Estimated as the concentration of indaziflam ‘before conjugation’ minus the concentration of indaziflam ‘after conjugation’. The reduction of indaziflam is presumed to represent that indaziflam that was successfully conjugated to the beads.

b Conjugation rate assuming a minimum number of bead binding sites, as specified by the manufacturer.

### Crude Root Protein Extraction

*A. thaliana* was germinated on square Petri dishes (15 × 100 × 100 mm^3^) containing 50 mL 1.5% agar (Neogen Food Safety, MI) and half-strength Murashige and Skoog medium (Caisson Laboratories, UT) were prepared. Seeds were surface-sterilized^24^ and sowed on the surface of solidified agar mixture. The Petri dishes were sealed with parafilm and incubated vertically in a growth chamber at 22 °C, 16-h light and 8-h dark, with the light intensity at 200 μmol m^–2^ s^–1^. After 1 week, plant roots were harvested and flash frozen in liquid nitrogen. *O. sativa* seeds were germinated in Petri dishes (diameter 9 cm) with filter paper moistened with distilled water, and incubated at 30 °C, 14 h light/10 h dark for 3 days before the root tissue was collected and stored in liquid nitrogen.

Root proteins were extracted from 400 mg fresh weight root tissue ground into powder in a mortar prechilled with liquid nitrogen. 1200 μL membrane protein solubilization buffer was added to the tissue and mixed well. The mixture was transferred into 1.5 mL centrifuge tubes and placed on ice. The samples were sonicated for 10 s at 50% amplitude at 4 °C. After the sonication, tubes were incubated on ice for 1 h, then centrifuged twice at 5000*g* for 5 min at 4 °C. The supernatant was transferred into new tubes as the root crude lysate.

### Preclear Protein Preparation and Precipitation

The crude protein lysate was added into quenched beads and incubated with continuous mixing on a rotisserie for 1 h at room temperature. About 200 μL protein lysate was precleared with 50 μL quenched beads slurry. After the incubation, the plant lysate was collected and used for pull-down. Indaziflam-conjugated and quenched beads (for negative control) were pipetted into 1.5 mL centrifuge tubes, respectively. The tubes were engaged into a magnetic stand, and the storage buffer was removed. The beads were washed with the wash/coupling buffer (PBS), and 200 μL precleared root lysate was added to each tube. The tubes were incubated with continuous mixing on a rotisserie for 2 h at room temperature. After the incubation, the protein solutions were removed, and the beads were washed twice with 50% DMSO in wash/coupling buffer (PBS). The beads were then resuspended with 60 μL 1× SDS-PAGE loading buffer, mixed gently and heated for 5 min at 95 °C to elute and denature the proteins. The supernatant was collected, and 5 μL was loaded and separated by SDS-PAGE (12%), together with the precleared protein lysate from each replicate. There were three replicates for each treatment. To prepare samples for liquid chromatography–mass spectrometry (LC-MS), 30 μL of protein samples above were loaded into a 5% SDS-PAGE gel, ran electrophoresis at 50 V for 15–20 min and stained with Coomassie Blue R-250 overnight. The bands were excised from the gel after being destained in 30% ethanol and 10% glacial acetic acid and stored in 5% acetic acid before liquid chromatography–mass spectrometry (LC-MS) identification.

### Liquid Chromatography with Tandem Mass Spectrometric (LC-MS/MS) Proteome Analysis

Gel bands were digested in-gel accordingly.^25^ Afterward, peptides were extracted from the gel by water bath sonication in a solution of 60% acetonitrile (ACN)/1% Trifluoroacetic acid (TFA) and vacuum-dried to about 2 μL. Samples were resuspended in 2% ACN/0.1% TFA to 20 μL. Two buffers were used hereafter: buffer A = 0.1% formic acid water solution; buffer B = 80% ACN and 20% buffer A. An injection of 5 μL sample was automatically made using a Thermo EASYnLC 1200 onto a Thermo Acclaim PepMap RSLC 0.1 mm × 20 mm C18 trapping column and washed for 5 min with buffer A. Bound peptides were then eluted over 35 min onto a Thermo Acclaim PepMap RSLC 0.075 mm × 250 mm resolving column with a gradient of 5–40% buffer B in 24 min, ramping to 90% at 25 min and held at 90% buffer B for the duration of the run at a constant flow rate of 300 mL min^–1^. Column temperature was maintained constant at 50 °C using an integrated column oven (PRSO-V2, Sonation GmbH, Biberach, Germany).

Eluted peptides were sprayed into a Thermo Scientific Q-Exactive HF-X mass spectrometer by using a FlexSpray spray ion source. Survey scans were taken in the Orbitrap (60 000 resolution, determined at *m*/*z* 200), and the top 15 ions in each survey scan were then subjected to automatic higher energy collision-induced dissociation with fragment spectra acquired at 15,000 resolution. The resulting MS/MS spectra were converted to peak lists using Mascot Distiller (v2.7.1) and searched against a protein database containing all *O. sativa japonica cv*. Nipponbare sequences were available from MSU Rice Genome Annotation Project (http://rice.uga.edu/) or the *A. thaliana* protein database available from The Arabidopsis Information Resource (www.arabidopsis.org; TAIR10) appended with common laboratory contaminants (downloaded from www.thegpm.org, cRAP project) using the Mascot searching algorithm (v2.7). The Mascot output was then analyzed by using Scaffold (v5.0.0) to probabilistically validate protein identifications. The mass spectrometric data sets have been deposited at the ProteomeXchange Consortium via the PRIDE partner repository (www.ebi.ac.uk/pride) in MIAPE-compliant format with the data set identifier PXD029589.

### Generation of the Candidate Protein List

With a list of identified proteins from each sample, we generated an enriched protein list for *A. thaliana* and *O. sativa*. The spectral counts of each protein (number of identified tandem MS/MS spectra matched to a given protein) were normalized by being divided by the total number of spectral counts in each replicate and averaged. Proteins showing a higher average spectral count number in the unbound flow-through fraction than the indaziflam-bound elution fraction were filtered out. After running one-tailed paired *t*-tests on the normalized spectral count number between the unbound flow-through and the indaziflam-bound elution samples, candidate proteins with *p*-values higher than 0.1 were filtered out.

Next, the candidate list was further shortened by looking for overlap between *A. thaliana* and *O. sativa*, using protein homology or gene ontology (GO). *A. thaliana* and *O. sativa* protein IDs were converted into the same format by retrieval from Uniprot (https://www.uniprot.org/). Using the Web site OrthoMCL DB (https://orthomcl.org/orthomcl/app/), these IDs were converted into ortholog groups and intersected with *A. thaliana*/*O. sativa* to generate corresponding protein IDs. Using expression information for *A. thaliana* (https://www.arabidopsis.org/) and *O. sativa* (https://ricexpro.dna.affrc.go.jp/), targets with very low expression in roots but high expression in other tissues were further filtered out. Analysis of these protein IDs was conducted based on their (i) level of enrichment identified by MS after pull-down, (ii) protein function, and (iii) putative biological process.

### Time-Course Observation of Endocytosis Using FM4-64

Four-day-old *A. thaliana* seedlings were treated individually with various chemicals. 8 μM indaziflam, 80 μM Dynasore (Adipogen Life Science, CA) a dynamin inhibitor, a positive control for Clathrin Mediated Endocytosis (CME) inhibition. Isoxaben, a known cellulose biosynthesis inhibitor and negative control, prepared at a final concentration of 100 μM in half-strength Murashige & Skoog (M519) basal medium (Caisson Laboratories, UT) in distilled water. The dynasore was used as positive control to test if indaziflam is inhibiting dynamin, which is one of candidate proteins from pull-down assay. The Isoxaben is included to test if indaziflam behaves similarly to the known herbicides in the same class. The seedlings were first incubated for 2 h in their respective treatments, after which 4 μM endocytic tracer FM4-64 SynaptoRed C2 (Biotium Inc., CA) was applied to each seedling for 5 min. Three washes in half-strength Murashige & Skoog (M519) basal medium and distilled water were performed to remove excess dye. Stained seedlings were slide-mounted immediately, and confocal imaging was performed at 5-, 20-, and 40 min poststaining to capture temporal dynamics of membrane internalization (i.e., vesicle endocytosis).

Confocal imaging was performed using an upright Olympus Fluoview spectral FV1000 confocal microscope with a UPLAPO 40× oil-immersion objective (NA 0.85). Fluorescence was excited with 10 mW solid state, 559 nm laser and detected in an epifluorescence channel (CHS1) using a 612 nm emission window and a DM405/488/559 dichroic mirror. Images were acquired as 1024 × 1024 pixel z-stacks with a lateral pixel size of 0.103 μm in *X* and *Y*- axis and a z-step of 0.84 μm and using Kalman line averaging. Fluorescence acquisition was conducted under nonsaturating pixel intensity conditions to ensure accurate quantification. FM4-64 fluorescence was visualized in the epidermal cells of the root tip, with images representing at least three independent biological replicates.

### Analysis and Quantification of Confocal Images

Quantitative analysis of fluorescence intensity was performed using FIJI^21^ (v2.16.0) to observe if the endocytosis decreases over time under various treatments as compared to control. Zone of division in the root tips was used for quantification. For each cell, a region immediately inside the plasma membrane was selected and raw fluorescence intensity was measured. Statistical analyses and visualization were conducted in R (v4.4.0).^25^ Pairwise statistical comparisons were conducted within each time point using two-tailed *t tests*. *p*-values were adjusted for multiple testing using the Bonferroni correction. A subset of treatment comparisons was selected a priori for evaluation, and only statistically significant comparisons with adjusted *p*-values ≤ 0.05 were retained. Data visualization was performed using ggplot2 ^26^ (v3.5.2) and Claude.^26^

### AlphaFold3­(AF3) Protein Structure Prediction with Indaziflam

The pulled-down proteins from *A. thaliana* and *O. sativa* (Table 2) were predicted *de novo* using AlphaFold3 ^20^ (AF3) with indaziflam (PubChem ID: 44146693) provided as a SMILES string. Protein sequences were obtained from NCBI using their accession identifiers. AF3 generated five independent models for each protein, with confidence metrics along with the predicted placement of indaziflam. All isoforms were included to generate five independent structures per sequence to assess potential interactions between indaziflam and each candidate protein. Root Mean Square Deviation (RMSD) values for indaziflam across the five replicates in each protein predicted were calculated using PyMOL (v3.0.5; Schrödinger Inc., NY) and an RMSD threshold of <6 Å was used as the cutoff for a reliable prediction. All plots were generated in R^27^ (v4.4.0) using ggplot2 ^28^ (v3.5.2).

**Table 2:** Candidate Proteins Enriched by Indaziflam Pull-Down from *Arabidopsis thaliana* and *Oryza sativa* Root Lysates

		*A. thaliana*			*O. sativa*		
group #	protein annotation	accessions	abundance in lysate^a^	enrichment (indaziflam/control)^a^	accessions	abundance in lysate^a^	enrichment (indaziflam/control)^a^
1	exocyst complex component 5	AT5G12370	0.00	10.02/0	LOC_Os12g13590	0.00	5.50/0
	clathrin heavy 1	AT3G11130	11.11	19.88/0	LOC_Os11g01380	21.32	70.33/14.66
	coatomer subunit β	AT4G31490	6.78	15.16/0	LOC_Os11g07280	12.45	18.62/6.07
	coatomer subunit γ	AT4G34450	11.45	36.44/10.36	LOC_Os07g10150	11.44	20.81/4.81
	coatomer subunit α	AT1G62020	1.87, 0.62	4.53/0, 3.14/0	LOC_Os03g50340	0.00	29.78/11.95
		AT2G21390					
	dynamin-related	AT1G14830	1.26	14.56/2.95	LOC_Os03g50520	9.79	21.14/4.85
	protein ROOT HAIR DEFECTIVE 3	AT3G13870	6.37	7.88/0.81	LOC_Os01g39310	12.20	6.62/2.33
2	PEP/phosphate translocator 1	AT5G33320	0.00	2.70/0	LOC_Os09g12600	0.00	2.19/0
	mitochondrial Rho GTPase	AT5G27540	3.98, 0	5.61/0, 2.70/0	LOC_Os03g59590	3.57	3.31/0
		AT3G63150					
	plasma membrane ATPase	AT4G30190	43.06	65.41/20.97	LOC_Os04g56160	14.94	71.95/25.04
3	histidine-tRNA ligase	AT3G02760	3.13	15.27/0	LOC_Os05g05840	2.32	5.50/1.22
	tryptophan-tRNA ligase	AT3G04600	5.57	7.76/3.54	LOC_Os12g35570	3.65	5.50/1.12
	proline-tRNA ligase	AT3G62120	7.32	10.46/6.22	LOC_Os12g25710	6.05	6.67/1.26
	eukaryotic translation initiation factor 3 subunit B	AT5G27640	2.85	13.61/1.88	LOC_Os10g41960	4.81	16.54/3.63
4	dolichyl-diphosphooligosaccharide-protein glycosyltransferase subunit 1	AT1G76400	1.23, 0.62	13.09/2.96, 5.18/0	LOC_Os05g23600	7.30	8.75/1.22
		AT2G01720					
	dolichyl-diphosphooligosaccharide-protein glycosyltransferase subunit 2	AT4G21150	9.16	38.94/8.15	LOC_Os01g68324	0.00	29.72/1.26
5	ABC transporter B	AT2G47000	1.87	11.29/3.31	LOC_Os03g54790	0.00	3.31/0
	cytochrome P450	AT2G40890	1.26, 6.31	8.10/0, 25.01/2.73	LOC_Os05g25640	17.34	36.45/4.81
		AT2G30490					
6	calcium-dependent protein kinase	AT1G50700	6.68, 0.98, 3.47, 4.00	11.33/3.22, 15.74/5.95, 16.85/7.89, 11.82/3.85	LOC_Os01g43410	6.22	7.74/0
		AT4G04720					
		AT3G20410					
		AT4G35310					
7	24-methylenesterol C-methyltransferase	AT1G76090	0.62	4.697/2.68	LOC_Os03g04340	5.97	12.17/2.38
	aminopeptidase M1	AT1G78570	10.93	22.19/6.44	LOC_Os08g30810	3.82, 7.55, 1.25	24.17/1.22, 14.3/7.14, 13.23/5.97
					LOC_Os02g12650		
					LOC_Os03g17000		
	UDP-glucose 6-dehydrogenase 4	AT5G39320	11.45	20.01/9.80	LOC_Os12g25690	36.92	39.65/30.96
	long chain acyl-CoA synthetase	AT4G05160, AT4G23850	8.06, 8.11	11.14/6.01, 26.18/1.88	LOC_Os11g04980	0.00	2.19/0
	phospho-2-dehydro-3-deoxyheptonate aldolase	AT4G39980	2.72	8.38/6.54	LOC_Os07g42960	20.48	31.80/12.13

a The unit for the numbers in the columns “Abundance in lysate” and “Enrichment (indaziflam/control)” is normalized spectral counts.

### Root Phenotyping of *A. thaliana* under Wortmannin and Indaziflam Treatment

*A. thaliana* seeds were surface-sterilized as described earlier and were grown on media supplemented with various chemicals, which were 0.1% (v/v) DMSO as controls, 400 pM of indaziflam (Bayer Crop Science, MO), 10 μM wortmannin (Adipogen Life Sciences, CA) and combination of 400 pM indaziflam and 10 μM wortmannin. The plants were grown in a growth chamber as described earlier and data were collected after 7 days. Roots were imaged on a Leica MZ16 stereo zoom microscope with a DFC295 color camera (Leica Microsystems, Wetzlar, Germany) using incident brightfield at 11.70× (1× objective, 0.73× C-mount adapter). Images were saved as uncompressed.tif files through Leica Application Suite. A scale bar was added using FIJI (v2.16.0)^21^

## Results

### Cellular Morphology of *A. thaliana* Treated with Indaziflam

Root phenotypes resulting from indaziflam treatment were examined to identify the anatomical changes associated with herbicide exposure. The zone of cell division of *A. thaliana* was morphologically similar between untreated and treated roots; however, characteristic cellulose biosynthesis inhibitor (CBI) phenotypes were evident in the elongation zone (Figure 2). In untreated roots, cells in the elongation zone elongated unidirectionally, forming elongated rectangular cells as they matured. In contrast, cells in the indaziflam-treated roots exhibited swelling and lacked directional elongation. As development progressed, cells frequently collapsed or burst and mature elongated cells failed to form (Figure 2).

**Figure 2:**
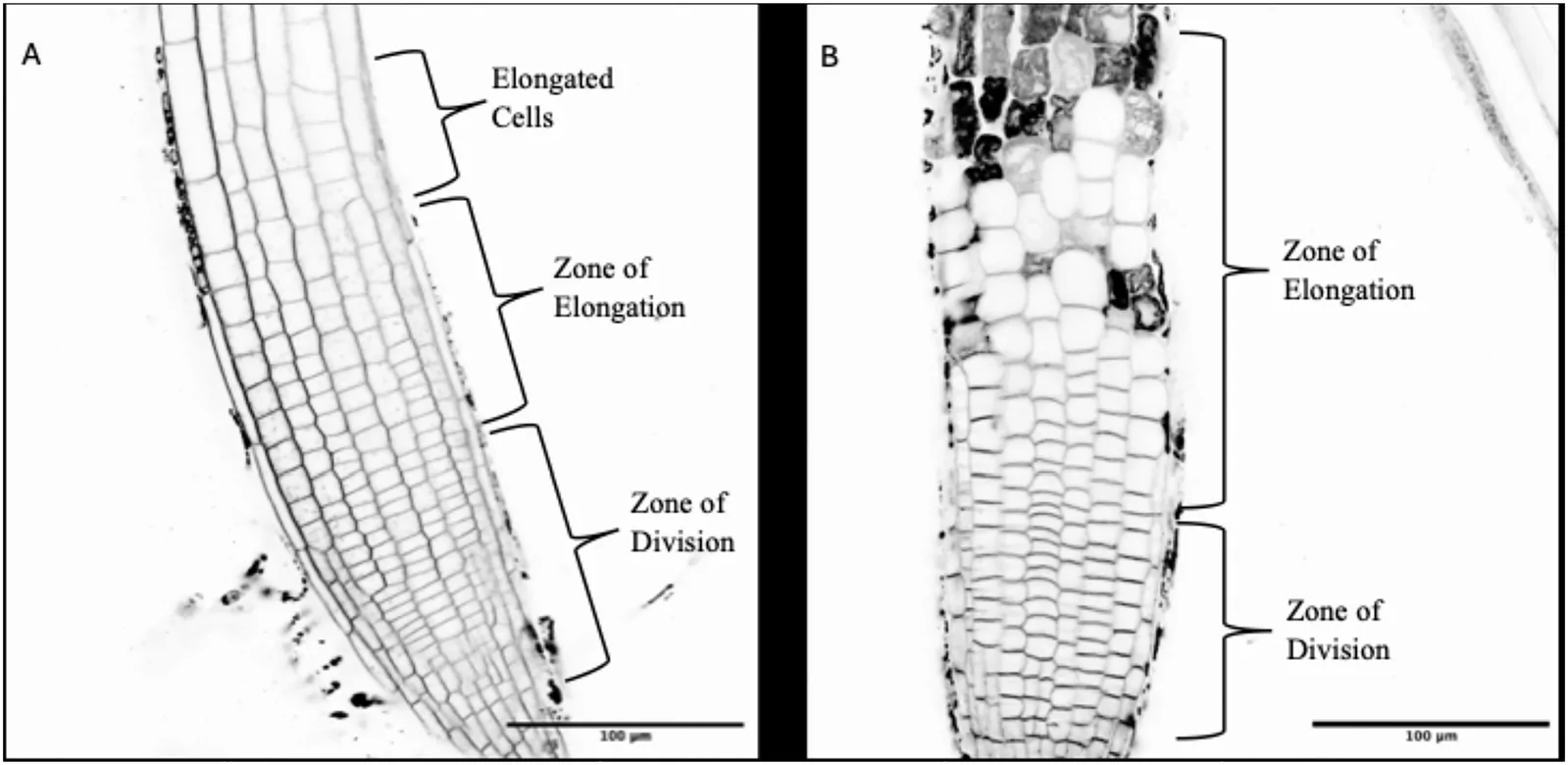
Root tips of 4-day-old *A. thaliana* (Col-0) seedlings treated with indaziflam for 2 h. Scale bar = 100 μm. (A) Roots with DMSO 0.1% (v/v) as control. We can see the zone of division and zone of elongation, with cells enlarging in size as they mature. (B) Indaziflam-treated roots, with characteristic symptomology of bulging of cells in the zone of elongation, which eventually burst due to cessation of expansion of the cell wall.

At the whole-root level, indaziflam treatment resulted in a dose-dependent reduction in the length of the root elongation zone (Figure 3A). Root hair density in the mature zone was markedly increased, although root hairs did not display obvious swelling. Primary roots exhibited a bumpy and curved surface, particularly at higher indaziflam concentrations (500–600 pM). Additionally, plants treated at 500 or 600 pM developed short, swollen lateral roots (Figure 3B), indicating substantial alterations in root system architecture.

**Figure 3:**
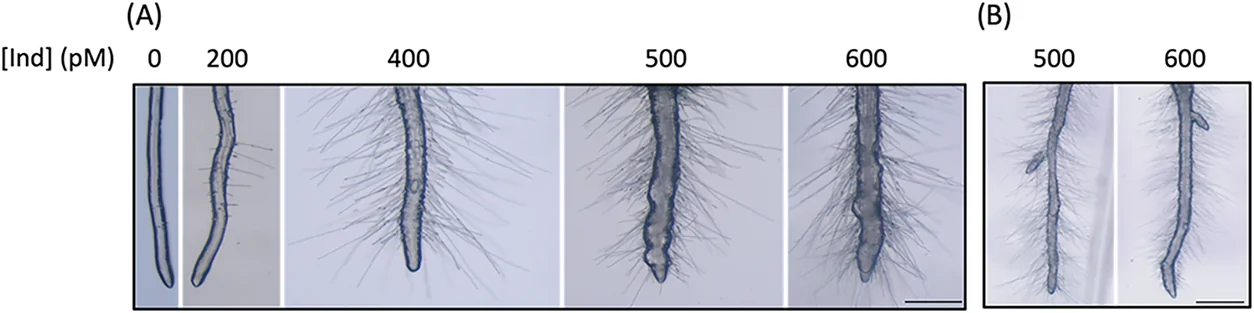
Root tips of *A. thaliana* (Col-0) seedlings treated with indaziflam for 12 days on 1.5% (w/v) agar-solidified half-strength Murashige and Skoog medium. Scale bar = 0.5 mm. (A) Elongation zone of the root is characterized by the absence of root hairs. A substantial elongation zone, as seen at 0 pM indaziflam, is greatly trimmed or completely eliminated with increasing concentrations of indaziflam. (B) Cessation of lateral root development at indaziflam concentration 500 pM (left) and 600 pM (right). Images were captured from the bottom of the plates through the medium using a Nikon stereomicroscope.

### Indaziflam Extinction Coefficient and Conjugation Efficiency

To identify potential indaziflam-binding proteins, pull-down assays were performed using root tissue lysates. The presence of a primary amine on indaziflam (Figure 1) enabled covalent coupling to NHS ester–functionalized magnetic beads. Prior to protein enrichment, conjugation efficiency was optimized and quantified. As shown in Figure S1, absorbance exhibited a strong linear relationship with indaziflam concentration in 100% DMSO (*R* ^2^ > 0.99). Using the calculated extinction coefficient (ε = 0.0044), the efficiency of indaziflam conjugation to NHS FlexiBind beads was determined.

According to the manufacturer’s specifications, the minimum concentration of reactive NHS functional groups on the beads is 3.4 mM. To estimate conjugation efficiency, indaziflam concentrations were measured before bead addition and after conjugation and bead removal. The decrease in indaziflam concentration corresponded to the amount of compound conjugated to the beads. Dividing this value by the reported minimum functional group concentration yielded the indaziflam conjugation rate (Table 1). Conjugation rates exceeding 100% in *Oryza sativa* samples indicate that the true molar concentration of reactive sites on the beads exceeded the manufacturer’s minimum specification of 3.4 mM. The consistently high conjugation efficiency demonstrates successful covalent linkage between indaziflam and NHS-ester magnetic beads, most likely via the primary amine group of indaziflam (Figure 1).

Following bead preparation, root lysates were generated from *A. thaliana* (dicot) and *O. sativa* (monocot), both of which are sensitive to indaziflam. A buffer containing a low concentration of nonionic detergent was used to gently solubilize membrane-associated proteins, allowing both soluble and hydrophobic proteins to be included as candidate interactors. Eluates from indaziflam-conjugated beads and negative control beads were separated by SDS-PAGE, revealing clear differences in protein abundance and composition between treatments (Figure 4). Protein abundance was substantially reduced in pull-down samples relative to that in the input lysate, consistent with selective protein enrichment. Proteins present in the eluates were identified by shotgun proteomics using LC-MS/MS, and candidates were defined by enrichment relative to that of negative controls. In total, 369 proteins from *A. thaliana* and 285 proteins from *O. sativa* were significantly enriched with indaziflam.

**Figure 4:**
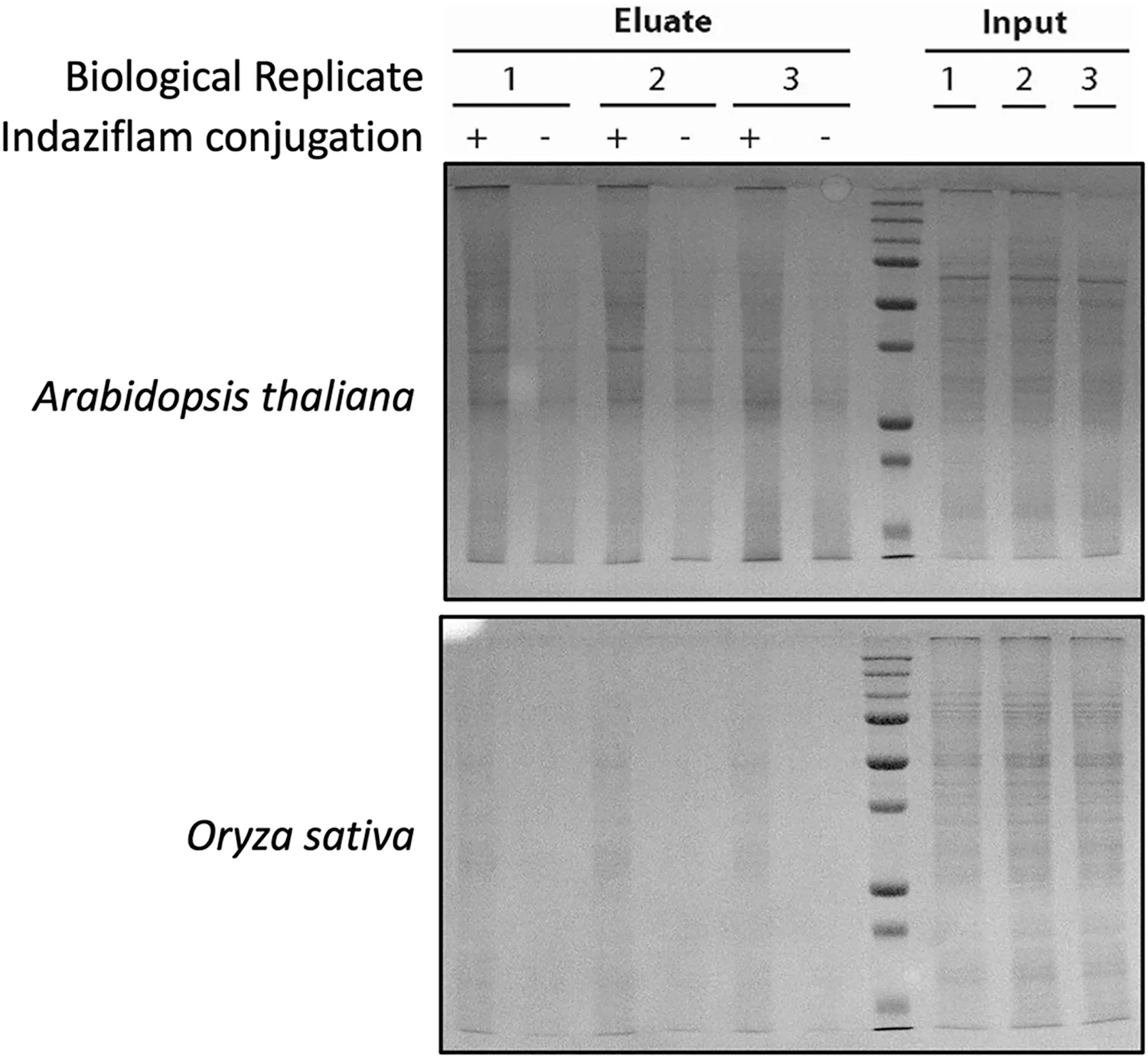
SDS-PAGE gel with eluted proteins from *A. thaliana* and *O. sativa*. Negative control elutes used unconjugated beads. The input (right) is solubilized total root lysate precleared with unconjugated beads.

Because both species are sensitive to indaziflam, enriched protein lists were cross-validated to identify conserved candidates using reciprocal best-hit BLAST analysis. This approach yielded a core set of conserved proteins comprising 50 from *A. thaliana* and 33 from *O. sativa*. These proteins were grouped into seven functional categories: (1) endocytosis/exocytosis, (2) energy supply, (3) protein translation, (4) glycosyltransferases, (5) nontarget-site resistance or metabolism, (6) phosphorylation, and (7) miscellaneous functions (Table 2). Proteins associated with endocytosis and exocytosis represented the largest functional category, with eight proteins from *A. thaliana* and seven from *O. sativa*. These proteins are known to participate in clathrin-mediated endocytosis,^29–31^ endoplasmic reticulum to Golgi vesicle-mediated transport^32^ and intra-Golgi vesicle-mediated transport.^33,34^

### Confocal Laser Scanning Microscopy and Quantification

Proteins associated with endocytosis and exocytosis constituted the largest functional category among indaziflam-enriched candidates, prompting a further examination of vesicle trafficking dynamics. FM4-64 staining combined with confocal laser scanning microscopy was used to monitor endocytosis in *A. thaliana* roots under an indaziflam treatment. Dynasore, a dynamin inhibitor, was used as a positive control for the inhibition of clathrin-mediated endocytosis (CME), while isoxaben, a cellulose biosynthesis inhibitor, served as a negative control.

FM4-64 initially labeled the plasma membrane and was internalized into intracellular vesicles within 5 min, appearing as punctate fluorescence (Figure 5). Control roots exhibited consistent dye internalization over a 40 min time course, with gradual signal reduction likely attributable to photobleaching (Figure 5A). Indaziflam-treated (Figure 5B) and isoxaben-treated (Figure 5D) roots displayed FM4-64 internalization over the observation period, whereas Dynasore treatment resulted in strong inhibition of CME (Figure 5C).

**Figure 5:**
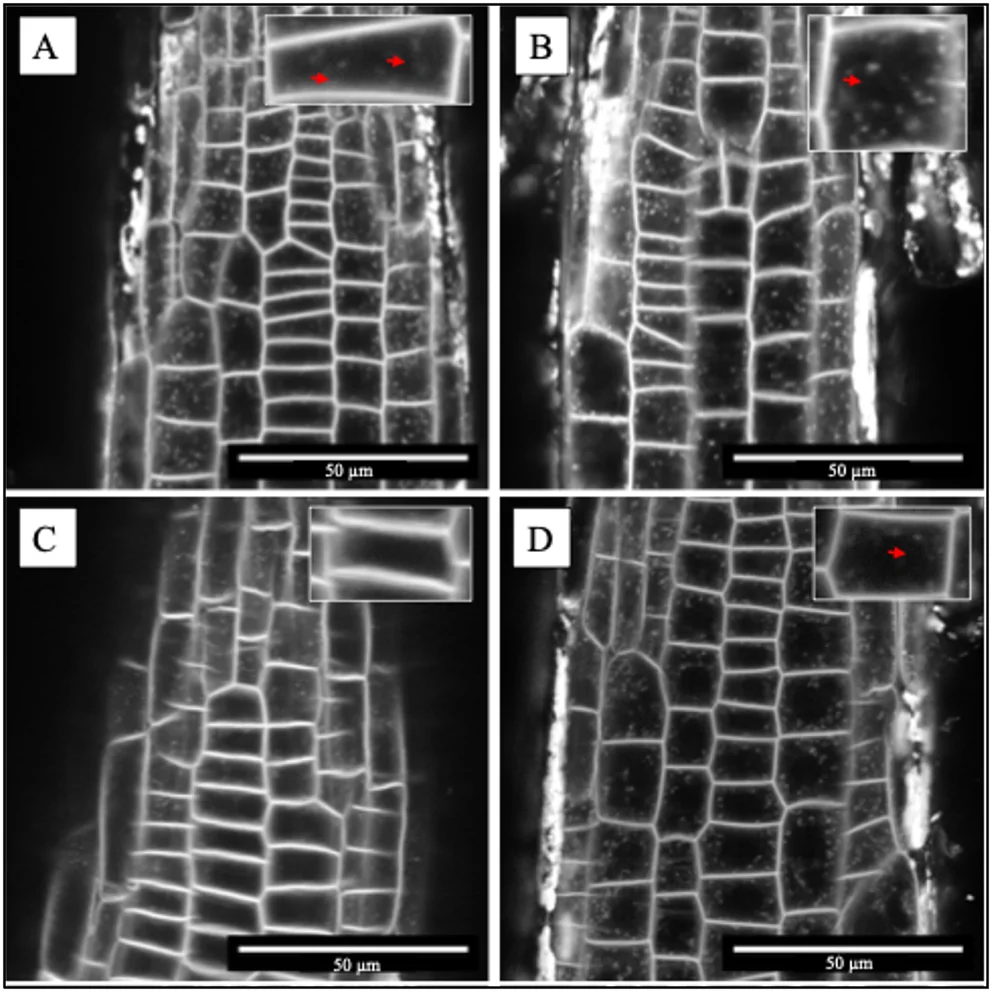
Quantification was performed on raw images with no postacquisition enhancements. 4-day-old *Arabidopsis thaliana* roots stained with FM4-64. (A) Treated with DMSO 0.001% concentration as a control. (B) Treated with 8 μM indaziflam, in DMSO 0.001% for 2 h. and stained with FM4-64. (C) Treated with 80 μM dynasore, in DMSO 0.001% for 3 h. (D) Treated with 100 μM isoxaben, for 2 h. All images are taken using a confocal microscope 40× objective. The images are enhanced using enhance contrast function of FIJI v2.16.0, using saturated pixel values are 0.35% and equalize histogram selection. Under control treatment (A), we see white pixels indicating properly endocytosed membrane vesicles stained with FM4-64 dye. The positive control of (C) dynasore treatment, which is endocytosis inhibitor, has no or significantly less endocytosis, as indicted by fewer white pixels in the cells.

At the 5 min time point, fluorescence intensity differed significantly between control and indaziflam-treated roots (adjusted *p* = 0.0499) and between control and dynasore-treated roots (adjusted *p* = 3.82 × 10^–20^), while no significant difference was observed between control and isoxaben treatments. At both 20 and 40 min time-points, dynasore-treated roots consistently retained significantly lower fluorescence than all other treatments (adjusted *p* < 0.001 for all pairwise comparisons), confirming sustained inhibition of endocytic uptake. By 40 min, fluorescence intensities in control, indaziflam, and isoxaben treatments converged, consistent with progressive FM4-64 internalization and saturation. Using indaziflam as reference, there was statistically differences in the fluorescence intensities at 5 min between indaziflam and isoxaben (adjusted *p* < 0.001), which are not present at 40 min time-point (Figure 6).

**Figure 6:**
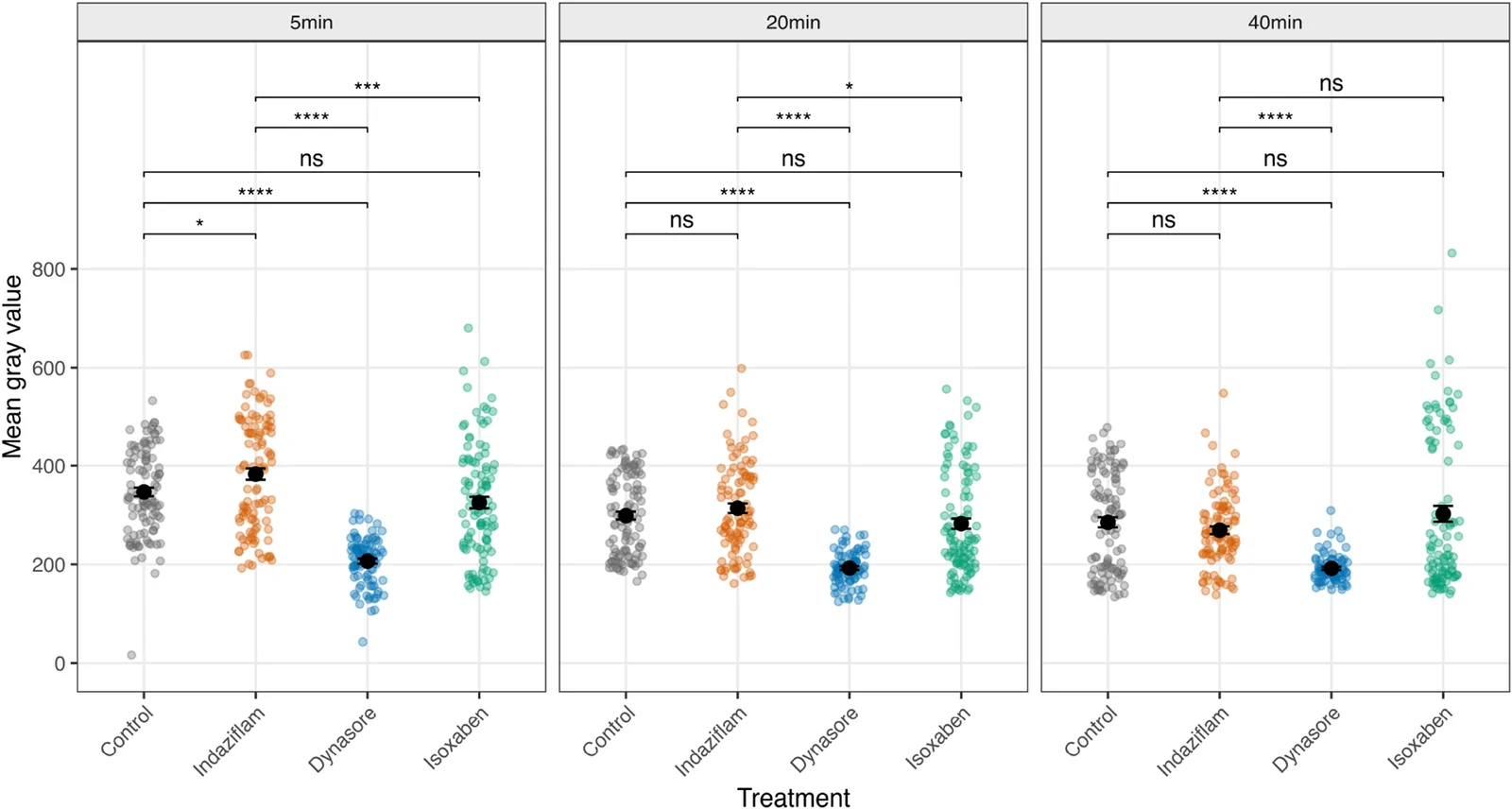
Fluorescence values of various treatments measured at 5, 20, and 40 min time-points of *A. thaliana* root cells under various treatments. *P*-value adjusted *p < 0.05, **p < 0.01, ***p < 0.001, ****p < 0.0001.

### AlphaFold3 Protein Modeling with Indaziflam

To test putative binding models for indaziflam, a total of 44 *O. sativa* and 55 *A. thaliana* protein–indaziflam complex models (including isomers) were generated using AlphaFold3. Prediction confidence was assessed using the root mean square deviation (RMSD) of indaziflam poses across five replicate predictions, with RMSD < 2 Å considered high confidence. Interface predicted template modeling (ipTM) scores were used to evaluate interface accuracy, while predicted template modeling (pTM) scores assessed global protein structure quality.

In *O. sativa*, ipTM scores ranged from 0.94 to 0.328 and pTM scores ranged from 0.94 to 0.374, with no steric clashes detected (Table S1). RMSD values ranged from 0.4914 to 42.872 Å. Among the modeled candidates, the lowest RMSD values were observed for calcium-dependent protein kinase, mitochondrial Rho GTPase, and aminopeptidase M1 (Figure S3). In contrast, trafficking related proteins, including coatomer subunits α and β, exhibited low confidence in indaziflam pose predictions.

For *A. thaliana*, ipTM values ranged from 0.93 to 0.336 and pTM values from 0.91 to 0.38, with no structural clashes detected (Table S1). RMSD values ranged from 0.4102 to 61.12 Å. The lowest RMSD predictions were observed for cytochrome P450 and several calcium-dependent protein kinases (Figure S4). Coatomer subunit β exhibited relatively stable predicted indaziflam poses compared with other coatomer subunits, whereas coatomer subunits α and γ showed lower confidence in ligand placement.

## Discussion

Understanding the specific binding site of herbicides is critically important for identifying herbicide resistance mechanisms, designing derivative chemistries, and explaining natural variation in selectivity and tolerance among species. Additionally, identifying a herbicide’s molecular target enhances our fundamental understanding of the roles of specific proteins and pathways in plant cell biology. Indaziflam was commercialized in 2010, and only limited cases of resistance have been reported to date. This likely reflects its restricted use patterns, primarily in orchards and turfgrass systems, where selection pressure is generally lower than in annual cropping systems. Recently, however, *Poa annua* (annual bluegrass) populations have been reported to exhibit both pre- and early postemergence resistance to indaziflam.^5^ Given that indaziflam has been one of the few highly effective options for controlling this species, the rapid emergence of resistance within little more than a decade is concerning. In our work, we made several significant methodological advances and discoveries toward understanding indaziflam’s SOA, ultimately supporting research on this molecule in the future.

First, for much of this work to be successful, an accurate determination of indaziflam concentrations is critical. We quantified the extinction coefficient of indaziflam in DMSO using the absorbance at 280 nm with high accuracy and repeatability, even at very low concentrations. This methodological advance is particularly valuable given that indaziflam is active at concentrations orders of magnitude lower than those of many other herbicides, making accurate dosage preparation challenging for future studies.

Second, our protein pull-down experiments surprisingly did not identify cellulose synthase (CESA) proteins as indaziflam binding partners in either *Arabidopsis thaliana* or *Oryza sativa*. This finding supports prior microscopy and biochemical studies suggesting that indaziflam is not a direct CESA inhibitor;^2^ however, several limitations must be considered. The abundance of cellulose synthase complexes (CSCs) in the tested tissues may have been insufficient for detection, and CSCs are membrane-embedded and can be unstable upon extraction from the plasma membrane.^35,36^ In addition, the pull-down assay enriches only for proteins that retain binding to indaziflam, while its primary amine is covalently linked to the beads. As a result, some indaziflam binding proteins may have escaped detection. An additional limitation of the pull-down assay is that the indaziflam was bound to the NHS magnetic beads via its primary free amine. If this primary amine plays an essential role in SOA binding, then this would greatly decrease the ability for us to bait the correct protein binding partner. We are also assuming indziflam is binding a SOA close to the protein surface as any deep binding pocket may be inaccessible due to the presence of the bead.

With that in mind, the analysis of proteins enriched from both *A. thaliana* and *O. sativa* revealed a notable enrichment of membrane-associated proteins with related cellular functions. Although membrane solubilization buffers were used, some proteins may have been copurified due to physical proximity on shared vesicles or membranes rather than through direct binding to indaziflam. Thus, it is likely that indaziflam binds to only a subset of the identified candidates. Among these, proteins involved in endocytosis and exocytosis are of particular interest, as the dynamic balance between these processes maintains CSC homeostasis at the plasma membrane.^31^ A working model for the involvement of candidate proteins in clathrin-mediated endocytosis (CME) of CSCs is presented in Figure 7. CESA proteins are synthesized in the endoplasmic reticulum, assembled into CSCs in the Golgi apparatus, and trafficked to the plasma membrane via the trans-Golgi network and microtubule-associated cellulose synthase compartments (MASCs/SmaCCs).^37,38^ After delivery, CSCs require approximately one minute to achieve steady motility at the plasma membrane.^38^ Removal of CSCs from the plasma membrane occurs via CME, specifically through adaptor protein complex 2 (AP2), likely to eliminate damaged, excess, or inactive complexes.^39,40^ Inhibition of CSC endocytosis, either pharmacologically or genetically via AP2 disruption, results in reduced root elongation and increased CSC density at the plasma membrane,^39^ phenotypes that partially resemble indaziflam treatment; however, distinct differences in root morphology suggest that indaziflam targets a unique protein or proteins within this pathway.

**Figure 7:**
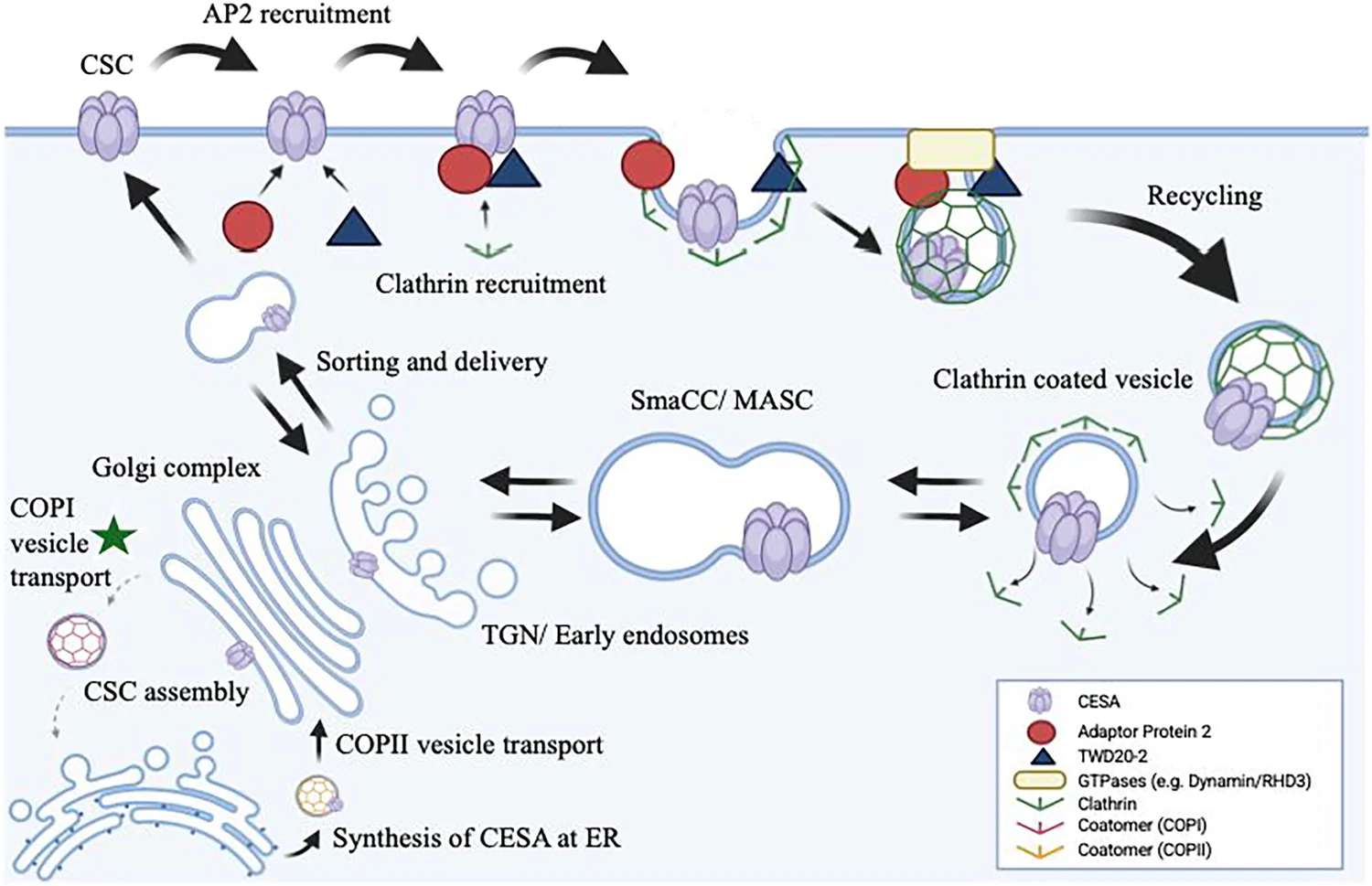
Proposed schematic of clathrin-mediated endocytosis for the repair or removal of damaged CESA from the plasma membrane and the key proteins that may be the site of action of indaziflam. Step 1: CESA becomes damaged and is recognized by AP2 and/or TWD40. Step 2: Clathrin is recruited to begin vesicle formation. Step 3: Vesicle evaginates. Step 4: GTPases (e.g., Dynamin or RHD3) are recruited to separate the vesicle from the plasma membrane. Step 5: GTPases and adaptor proteins etc. are removed after the scission. Step 6: Clathrin skeleton dissociates from the vesicle. Step 7: Vesicle fuses with the early endosome. Step 8: Broken CESA enters the retrograde trans-Golgi network. Step 9: Repaired CESA is sent back to work on the plasma membrane. Step 10: Golgi to ER retrograde vesicles are formed on the cis surface of the Golgi utilizing coatomers (Specifically COPI) and GTPase. Step 11: Retrograde vesicles fuse with the ER. Star represents hypothesized target site of indaziflam as COP1 assembly.

CSC homeostasis likely depends on tightly regulated signaling cues that determine whether complexes are delivered to, retained at, or removed from the plasma membrane. Disrupting this balance is often lethal to plant cells, as demonstrated in *A. thaliana* mutants. Fluorescently labeled CESA6 in indaziflam-treated *A. thaliana* exhibits two characteristic phenotypes: reduced average CSC velocity and marked accumulation at the plasma-membrane focal plane. These phenotypes closely resemble those observed in AP2 knockout mutants.^39^ Inhibition of CME components such as AP2 or the GTPase dynamin prevents CSC internalization, leading to accumulation of both functional and defective CSCs at the plasma membrane.^41^ The presence of damaged or dissociated complexes likely contributes to the observed reduction in the average CSC velocity.

Confocal microscopy using FM4-64 indicated that indaziflam is not a complete inhibitor of CME (Figure 5). One possible explanation is that indaziflam targets proteins involved in interorganelle trafficking, such as coatomer subunits (α, β, or γ). Inhibition of coatomers would not be readily detected by FM4-64 assays, as interorganelle trafficking contributes only modestly to the overall endocytic signal. Furthermore, the absence of synergistic effects between indaziflam and wortmannin (a strong inhibitor of vacuolar trafficking) supports the conclusion that indaziflam disrupts a pathway distinct from wortmannin-sensitive endocytosis.^42^ Combined treatments produced additive rather than synergistic root phenotypes, indicating partially independent modes of action (Figure S2).

In addition to CME-related proteins, other functional groups identified in the pull-down assays may be linked to CSC dynamics or indaziflam-induced root phenotypes. For example, Rab-H1b, a Golgi-localized GTPase, regulates trafficking and plasma membrane distribution of CESA6.^43^ A plasma-membrane-localized ATPase identified in the pull-down may also be relevant, as vacuolar ATPases have been shown to play essential roles in vesicle trafficking during cellulose synthesis and cell expansion.^44^ Furthermore, DYNAMIN-LIKE PROTEAN 1C (DRP1C) is known to colocalize with clathrin light chain 2,^30^ further implicating endocytic pathways.

AlphaFold3 modeling of indaziflam interactions with candidate proteins identified coatomer subunit β as a potential binding partner with a consistently stable indaziflam pose in *A. thaliana*. Although AlphaFold predictions are biased toward proteins with known small-molecule ligands (with coatomers being structural proteins lacking native ligands), the predicted stability of indaziflam-binding suggests a plausible interaction site. These results also raise the possibility that ATPases or GTPases involved in vesicle trafficking may represent primary indaziflam targets. Binding a structural protein is not unheard of; Group 3 herbicides, such as trifluralin, disrupt the interaction of β-tubulin and α-tubulin and therefore the proper assembly and disassembly of microtubules.^45^ Top candidates predicted by AlphaFold also include calcium kinases and tRNA-ligases. AlphaFold is likely biased toward proteins such as these that have known binding sites that interact with small molecules.

Root phenotypes provide additional clues regarding indaziflam’s SOA. Abnormal root growth patterns similar to those induced by indaziflam have been observed in numerous *A. thaliana* mutants defective in CSC or CESA-associated pathways. For example, mutations in *ROOT HAIR DEFECTIVE 3* (RHD3) result in reduced root length and aberrant root hair morphology.^46^ In contrast, indaziflam-treated plants displayed straight but densely packed root hairs, suggesting mechanistic overlap but a distinct molecular target. Despite extensive screening of EMS mutant libraries, others have not identified strongly indaziflam-resistant mutants, suggesting that point mutations conferring resistance may be lethal or highly detrimental to seed development.

To our knowledge, this study represents the first attempt to identify indaziflam-binding proteins using a ligand-baited pull-down approach rather than forward mutagenesis. Our findings support the hypothesis that indaziflam inhibits one or more proteins involved in CSC trafficking or translocation, thereby disrupting proper cell wall assembly. Future studies employing yeast two-hybrid assays, bimolecular fluorescence complementation, and coimmunoprecipitation will be critical for validating specific indaziflam–protein interactions and refining its molecular site of action.

## Supplementary Material



## Notes

Figure S1: Absorbance of indaziflam in 100% DMSO at 280 nm. Figure S2: Phenotyping of the *A. thaliana* with indaziflam and wortmannin treatments. Figures S3 and S4: Alphafold3 modeling scores of the pull-down proteins with indaziflam. Table S1: AF3 confidence metrics for the protein models predicted using indaziflam as ligand. (pdf)

## Abbreviations

CSC: cellulose synthase complex
CME: clathrin-mediated endocytosis
ipTM: interface predicted template modeling
pTM: predicted template modeling
