# Supplementary material for: Ligand-Baited Proteomics Reveals Vesicle Trafficking Proteins as Putative Site of Action of Indaziflam
Source: J Agric Food Chem. 2026 Oct 9;74(41):32184–94. doi: 10.1021/acs.jafc.6c07779 (PMC13693455; doi:10.1021/acs.jafc.6c07779)
Supplement: Supplementary file 1 [file jf6c07779_si_001.pdf]

Title: Ligand-baited proteomics reveals vesicle trafficking proteins as putative site of action of indaziflam

Authors: Mohit Mahey[1,3], Peter Knut Lundquist[2,3,*], Eric Lloyd Patterson[1,*]

[1]Department of Plant, Soil, and Microbial Sciences, Michigan State University, East Lansing, MI 48824, USA

[2]Department of Biochemistry & Molecular Biology, Michigan State University, East Lansing, MI 48824, USA

[3]Plant Resilience Institute, Michigan State University, East Lansing, MI 48824, USA

*Co-corresponding authors:

- Eric L. Patterson. Department of Plant, Soil, and Microbial Sciences, Michigan State University, East Lansing, MI 48824, USA. Email: patte543@msu.edu. Ph: +1 517 353 0467
- Peter K. Lundquist. Email: pklundqu@msu.edu. Ph: +1 517 353 0441

ORCIDs:

Mohit Mahey (0009-0009-5479-7995)

Peter Knut Lundquist (0000-0001-8390-8089)

Eric Lloyd Patterson (0000-0001-7111-6287)

Supplementary Figure 1. Absorbance of indaziflam in 100% DMSO at 280 nm. The dotted line is the linear trendline for indaziflam Ɛ.

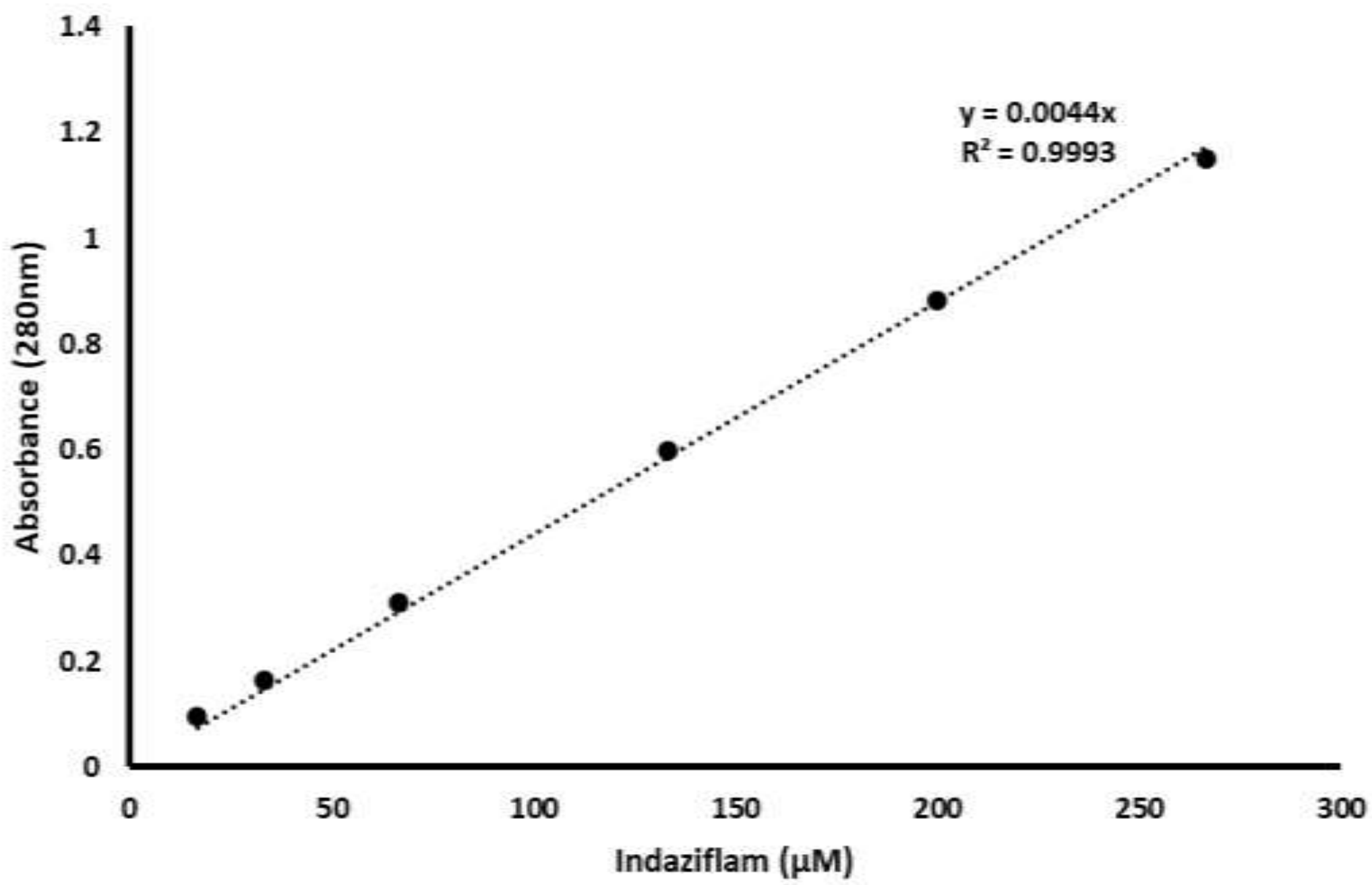

Supplemental Figure 2. Root phenotype of the *A. thaliana* after 7 days of treatment grown on agar 1.5% (w/v) and half strength Murashige and Skoog media. Scale bar = 1 mm. (A) *A. thaliana* roots as control (B) 400 pM indaziflam treated (C) 10 µM wortmannin (D) combination of 400 pM indaziflam and 10 µM wortmannin. Below the picture are the close-ups of the root tips of each corresponding plant. (n=10)

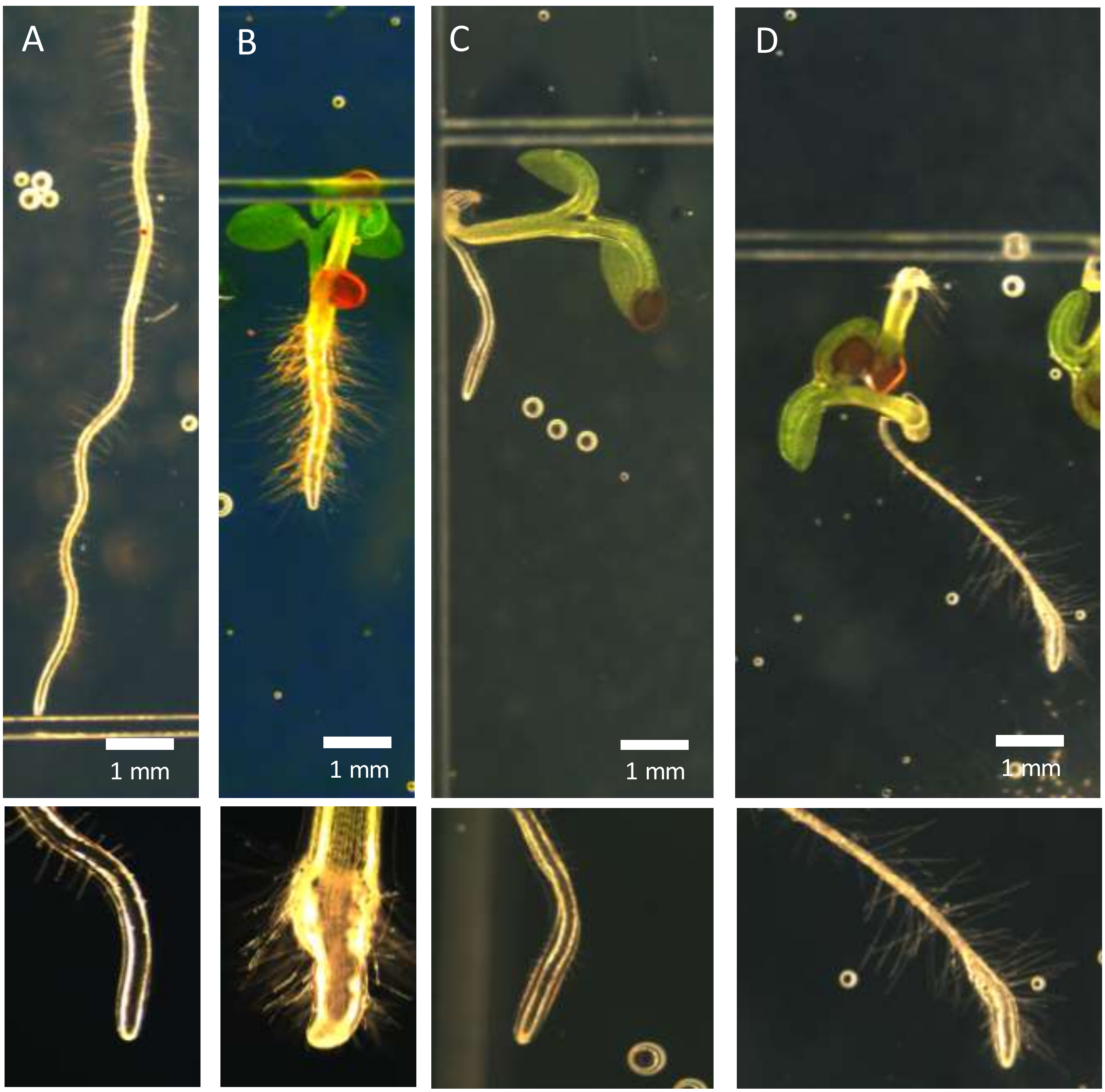

Supplemental Figure 3. AlphaFold3-predicted indaziflam binding pose across *Oryza sativa* pulldown proteins. Boxplots show the distribution of predicted ligand RMSD (Å) for indaziflam across AF3 seed models for each O. sativa protein. Lower RMSD values indicate a more stable and reproducible ligand pose across seeds. Proteins are ordered left to right by increasing mean RMSD. The red dashed line marks the 2 Å threshold frequently used to define highly stable predicted ligand positioning.

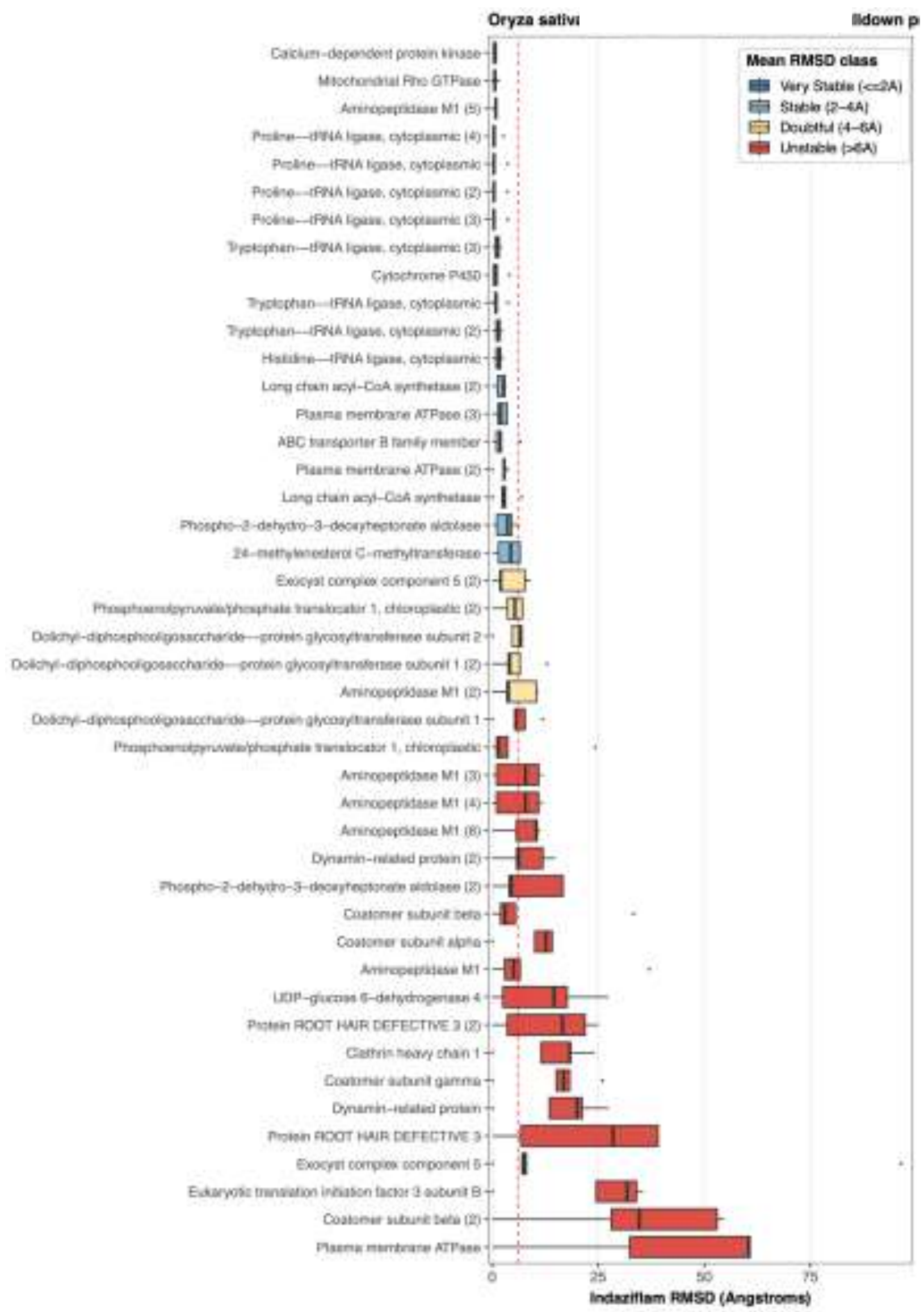

Supplemental Figure 4. AlphaFold3-predicted indaziflam binding stability across *Arabidopsis thaliana* pulldown proteins. Boxplots represent the distribution of indaziflam ligand RMSD (Å) across 20 AF3 seed models for each A. thaliana protein. Proteins are arranged from lowest to highest mean RMSD to visualize stability trends. The horizontal dashed line marks the 2 Å stability cutoff.

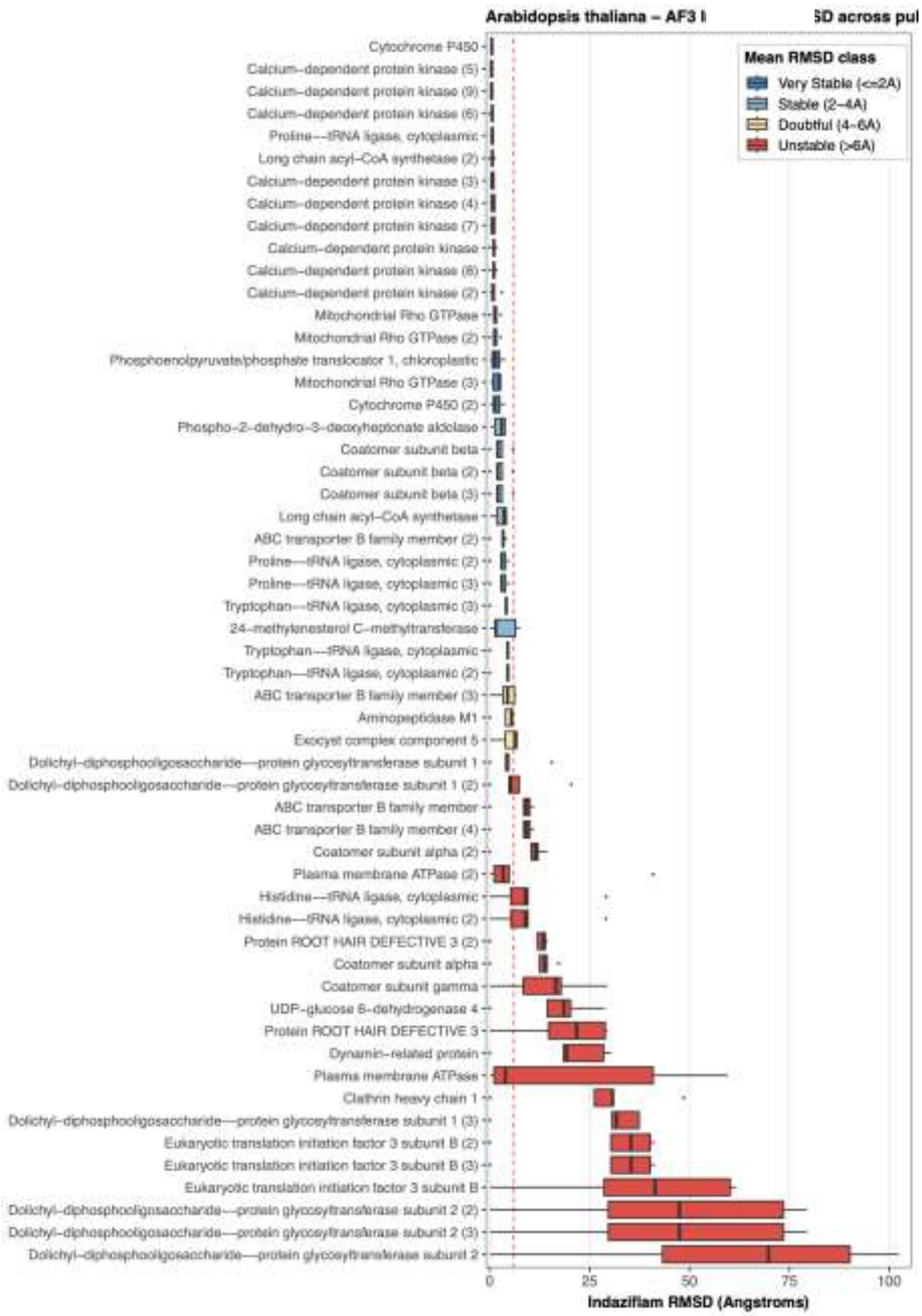

Supplemental Table 1. AF3 confidence metrics for the protein models predicted using indaziflam as ligand

Group No	Protein annotation	*A. thaliana* Accessions	Average iPTM	SD iPTM	Average pTM	Average Fraction Disordered	*O. sativa* Accessions	Average iPTM	SD iPTM	Average pTM	Average Fraction Disordered
1	Exocyst complex component 5	BAH56814.1	0.550	0.025	0.498	0.238	LOC_Os12g13590.2	0.696	0.024	0.818	0.024
							LOC_Os12g13590.1	0.596	0.030	0.670	0.056
	Clathrin heavy chain 1	NP_187724.2	0.364	0.027	0.380	0.050	LOC_Os11g01380.1	0.406	0.037	0.374	0.050
	Coatomer subunit beta	NP_001329405.1	0.562	0.036	0.612	0.084	LOC_Os11g07280.1	0.612	0.073	0.558	0.074
		NP_001329406.1	0.562	0.036	0.612	0.084	LOC_Os11g07280.2	0.394	0.121	0.568	0.104
		NP_194877.2	0.562	0.036	0.612	0.084					
	Coatomer subunit gamma	NP_195171.2	0.524	0.068	0.568	0.062	LOC_Os07g10150.1	0.434	0.060	0.540	0.066
	Coatomer subunit alpha	NP_176393.1	0.400	0.048	0.508	0.082	LOC_Os03g50340.1	0.328	0.060	0.456	0.112
		NP_179734.1	0.400	0.046	0.506	0.086					
	Dynamin-related protein	NP_172936.1	0.694	0.070	0.698	0.040	LOC_Os03g50520.2	0.638	0.058	0.542	0.032
							LOC_Os03g50520.1	0.576	0.080	0.680	0.030
	Protein ROOT HAIR DEFECTIVE 3	NP_188003.1	0.670	0.044	0.654	0.132	LOC_Os01g39310.1	0.780	0.090	0.676	0.150
		NP_974308.1	0.622	0.029	0.642	0.162	LOC_Os01g39310.3	0.692	0.037	0.742	0.066
2	Phosphoenolpyruvate/phosphate translocator 1, chloroplastic	NP_198317.1	0.860	0.014	0.720	0.260	LOC_Os09g12600.1	0.756	0.055	0.734	0.204
							LOC_Os09g12600.2	0.728	0.063	0.664	0.248
	Mitochondrial Rho GTPase	NP_567139.1	0.930	0.000	0.820	0.036	LOC_Os03g59590.1	0.888	0.011	0.860	0.050
		NP_001031953.1	0.882	0.008	0.812	0.050					
		NP_198106.1	0.882	0.008	0.812	0.050					
	Plasma membrane ATPase	NP_194748.1	0.592	0.086	0.784	0.062	LOC_Os04g56160.2	0.776	0.017	0.644	0.128
							LOC_Os04g56160.1	0.588	0.044	0.776	0.052
		NP_001190870.1	0.566	0.055	0.750	0.066	LOC_Os04g56160.3	0.588	0.020	0.788	0.052
3	Histidine--tRNA ligase, cytoplasmic	NP_001325800.1	0.622	0.113	0.562	0.020	LOC_Os05g05840.1	0.734	0.021	0.666	0.040
		NP_186925.4	0.622	0.113	0.562	0.020					
	Tryptophan--tRNA ligase, cytoplasmic	NP_001078104.1	0.760	0.029	0.890	0.034	LOC_Os12g35570.1	0.808	0.011	0.890	0.054
		NP_187110.1	0.760	0.029	0.890	0.034	LOC_Os12g35570.2	0.754	0.005	0.730	0.110
		NP_974219.1	0.760	0.029	0.890	0.032	LOC_Os12g35570.3	0.748	0.019	0.742	0.108
	Proline--tRNA ligase, cytoplasmic	NP_001326276.1	0.828	0.008	0.884	0.070	LOC_Os12g25710.1	0.782	0.020	0.886	0.054
							LOC_Os12g25710.2	0.782	0.020	0.886	0.054

		NP_191771.1	0.766	0.009	0.860	0.090	LOC_Os12g25710.3	0.782	0.020	0.886	0.054
		NP_850736.1	0.766	0.009	0.860	0.090	LOC_Os12g25710.4	0.616	0.011	0.696	0.060
	Eukaryotic translation initiation factor 3 subunit B	NP_001031954.1	0.602	0.129	0.632	0.094	LOC_Os10g41960.1	0.510	0.048	0.632	0.090
		NP_001330567.1	0.546	0.102	0.648	0.102					
		NP_568498.1	0.546	0.102	0.648	0.102					
4	Dolichyl-diphosphooligosaccharide–protein glycosyltransferase subunit 1	NP_178281.1	0.720	0.056	0.856	0.080	LOC_Os05g23600.2	0.750	0.062	0.798	0.100
		NP_001322656.1	0.672	0.100	0.706	0.042	LOC_Os05g23600.1	0.610	0.056	0.660	0.054
		NP_177766.1	0.620	0.072	0.660	0.040					
	Dolichyl-diphosphooligosaccharide–protein glycosyltransferase subunit 2	NP_001190784.2	0.422	0.069	0.678	0.128	LOC_Os01g68324.3	0.678	0.023	0.758	0.024
		NP_001190785.1	0.336	0.084	0.678	0.120					
		NP_193847.2	0.336	0.084	0.678	0.120					
5	ABC transporter B family member	NP_001323913.1	0.776	0.021	0.708	0.138	LOC_Os03g54790.1	0.694	0.0167	0.752	0.084
		NP_001323915.1	0.776	0.022	0.702	0.110					
		NP_001323912.1	0.708	0.042	0.732	0.108					
		NP_001323916.1	0.708	0.042	0.732	0.108					
	Cytochrome P450	NP_180607.1	0.862	0.004	0.910	0.032	LOC_Os05g25640.1	0.816	0.009	0.910	0.050
		NP_850337.1	0.784	0.029	0.898	0.028					
6	Calcium-dependent protein kinase	NP_001327803.1	0.926	0.005	0.620	0.152	LOC_Os01g43410.1	0.940	0.000	0.642	0.094
		NP_188676.1	0.926	0.005	0.620	0.152					
		NP_001320604.1	0.922	0.004	0.690	0.142					
		NP_001329080.1	0.920	0.000	0.636	0.140					
		NP_195257.1	0.920	0.000	0.636	0.140					
		NP_175485.1	0.916	0.005	0.640	0.120					
		NP_001320603.1	0.914	0.005	0.628	0.112					
		NP_001319867.1	0.874	0.009	0.624	0.128					
		NP_192381.1	0.874	0.009	0.624	0.128					
7	24-methylenesterol C-methyltransferase	NP_177736.1	0.690	0.034	0.752	0.038	LOC_Os03g04340.1	0.762	0.018	0.742	0.098
	Aminopeptidase M1	NP_177978.1	0.638	0.016	0.510	0.070	LOC_Os03g17000.2	0.892	0.008	0.530	0.066
							LOC_Os03g17000.3	0.892	0.008	0.530	0.066
							LOC_Os03g17000.4	0.832	0.008	0.624	0.074
							LOC_Os02g12650.2	0.684	0.057	0.930	0.000
							LOC_Os02g12650.1	0.680	0.057	0.938	0.000
							LOC_Os08g30810.1	0.680	0.031	0.940	0.000
	UDP-glucose 6-dehydrogenase 4	NP_198748.1	0.470	0.037	0.856	0.000	LOC_Os12g25690.1	0.486	0.072	0.846	0.000
	Long chain acyl-CoA synthetase	NP_192425.1	0.756	0.036	0.864	0.000	LOC_Os11g04980.1	0.758	0.008	0.816	0.046

		NP_194116.1	0.676	0.015	0.810	0.000	LOC_Os11g04980.2	0.736	0.009	0.828	0.050
	Phospho-2-dehydro-3-deoxyheptonate aldolase	NP_195708.1	0.558	0.013	0.788	0.140	LOC_Os07g42960.1	0.574	0.005	0.780	0.170
							LOC_Os07g42960.2	0.448	0.081	0.732	0.036
